# How the weather affects the pain of citizen scientists using a smartphone app

**DOI:** 10.1038/s41746-019-0180-3

**Published:** 2019-10-24

**Authors:** William G. Dixon, Anna L. Beukenhorst, Belay B. Yimer, Louise Cook, Antonio Gasparrini, Tal El-Hay, Bruce Hellman, Ben James, Ana M. Vicedo-Cabrera, Malcolm Maclure, Ricardo Silva, John Ainsworth, Huai Leng Pisaniello, Thomas House, Mark Lunt, Carolyn Gamble, Caroline Sanders, David M. Schultz, Jamie C. Sergeant, John McBeth

**Affiliations:** 1 Centre for Epidemiology Versus Arthritis, Manchester Academic Health Science Centre, The University of Manchester, Manchester, UK; 2 Health eResearch Centre, Manchester Academic Health Science Centre, The University of Manchester, Manchester, UK; 3 NIHR Greater Manchester Biomedical Research Centre, Manchester Academic Health Science Centre, The University of Manchester, Manchester, UK; 4 Department of Public Health Environments and Society, London School of Hygiene & Tropical Medicine, London, UK; 5 Centre for Statistical Methodology, London School of Hygiene & Tropical Medicine, London, UK; 6 IBM Research, Haifa, Israel; 7 uMotif Limited, London, UK; 8 Department of Anesthesiology, Pharmacology and Therapeutics, University of British Columbia, Vancouver, BC Canada; 9 Department of Statistical Science, UCL London, UK; 10 Alan Turing Institute, London, UK; 11 Discipline of Medicine, The University of Adelaide, Adelaide, Australia; 12 School of Mathematics, The University of Manchester, Manchester, UK; 13 IBM Research, Hartree Centre, Sci-Tech, Daresbury, UK; 14 Greater Manchester Patient Safety Translational Research Centre, The University of Manchester, Manchester, UK; 15 NIHR School of Primary Care, Manchester Academic Health Science Centre, The University of Manchester, Manchester, UK; 16 Centre for Atmospheric Science, School of Earth and Environmental Sciences, The University of Manchester, Manchester, UK; 17 Centre for Biostatistics, Manchester Academic Health Science Centre, The University of Manchester, Manchester, UK

**Keywords:** Risk factors, Chronic pain, Epidemiology

## Abstract

Patients with chronic pain commonly believe their pain is related to the weather. Scientific evidence to support their beliefs is inconclusive, in part due to difficulties in getting a large dataset of patients frequently recording their pain symptoms during a variety of weather conditions. Smartphones allow the opportunity to collect data to overcome these difficulties. Our study *Cloudy with a Chance of Pain* analysed daily data from 2658 patients collected over a 15-month period. The analysis demonstrated significant yet modest relationships between pain and relative humidity, pressure and wind speed, with correlations remaining even when accounting for mood and physical activity. This research highlights how citizen-science experiments can collect large datasets on real-world populations to address long-standing health questions. These results will act as a starting point for a future system for patients to better manage their health through pain forecasts.

## Introduction

Weather has been thought to affect symptoms in patients with chronic disease since the time of Hippocrates over 2000 years ago.^1^ Around three-quarters of people living with arthritis believe their pain is affected by the weather.^2,3^ Many report their pain is made worse by the cold, rain, and low atmospheric pressure. Others report that their pain is made worse by warmth and high humidity. Despite much research examining the existence and nature of the weather–pain relationship,^4^ there remains no scientific consensus. Studies have failed to reach consensus in part due to their small sample sizes or short durations (commonly fewer than 100 participants or one month or less); by considering a limited range of weather conditions; and heterogeneity in study design (e.g. the populations studied, methods for assessing pain, assumptions to determine the weather exposure, and statistical analysis techniques).^5–11^ Resolving this question requires collection of high-quality symptom and weather data on large numbers of individuals. Such data also need to include other factors potentially linked to daily pain variation and weather, such as mood and amount of physical activity. Collecting this kind of multi-faceted data in large populations over long periods of time, however, has been difficult.

The increasing uptake of smartphones offers new and significant opportunities for health research.^12^ Smartphones allow the integration of data collection into daily life using applications (apps). Furthermore, embedded technologies within the smartphones, such as the Global Positioning System (GPS), can be used to link the data collection to specific locations. We created Cloudy with a Chance of Pain,^13,14^ a national United Kingdom smartphone study, to collect a large dataset to examine the relationship between local weather and daily pain in people living with long-term pain conditions.

## Results

### Recruitment and retention

**Fig. 1:**
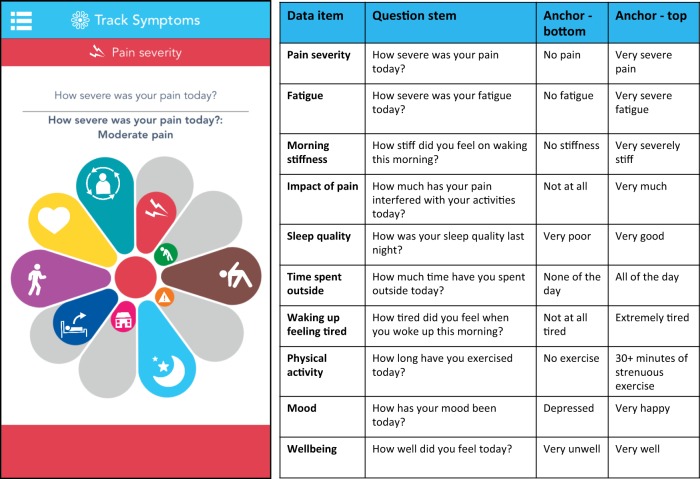
User interface of the study app (uMotif, London). Each colored segment represents one of the ten data items. Participants report their symptoms on a five-point scale by dragging the segment from the center outwards

**Fig. 2:**
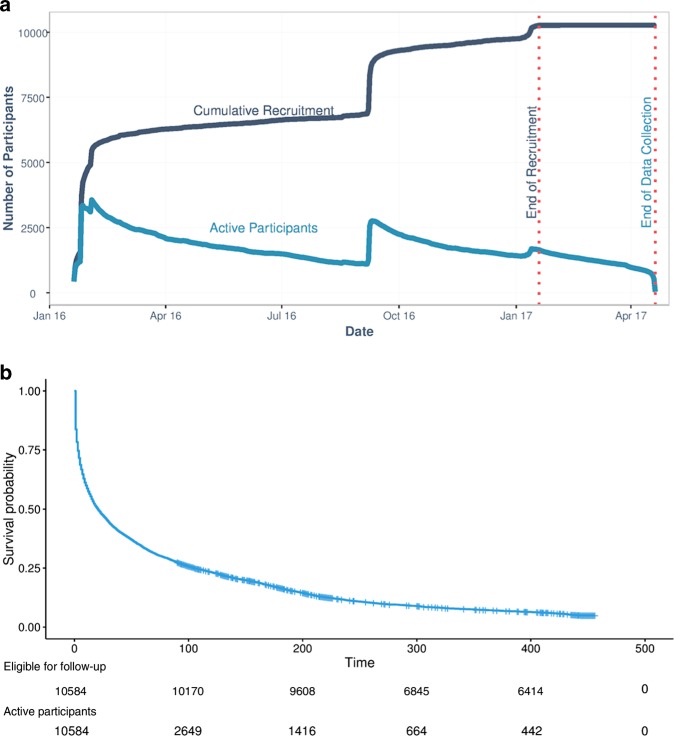
Recruitment and retention. **a** Cumulative recruitment and number of active participants through time. The blue line represents the cumulative number of participants with a completed baseline questionnaire and at least one pain score submitted. The red line represents the current number of active participants (i.e. those who have submitted their first but not yet their last pain score in the study period). **b** Retention through time. The graph represents the retention of active participants through time as a survival probability from the day of their recruitment. Participants were censored when they were no longer eligible for follow-up. Eligible follow-up time ranged from 90 days (for those recruited on 20 January 2017) to 456 days (for those recruited on 20 January 2016)

**Fig. 3:**
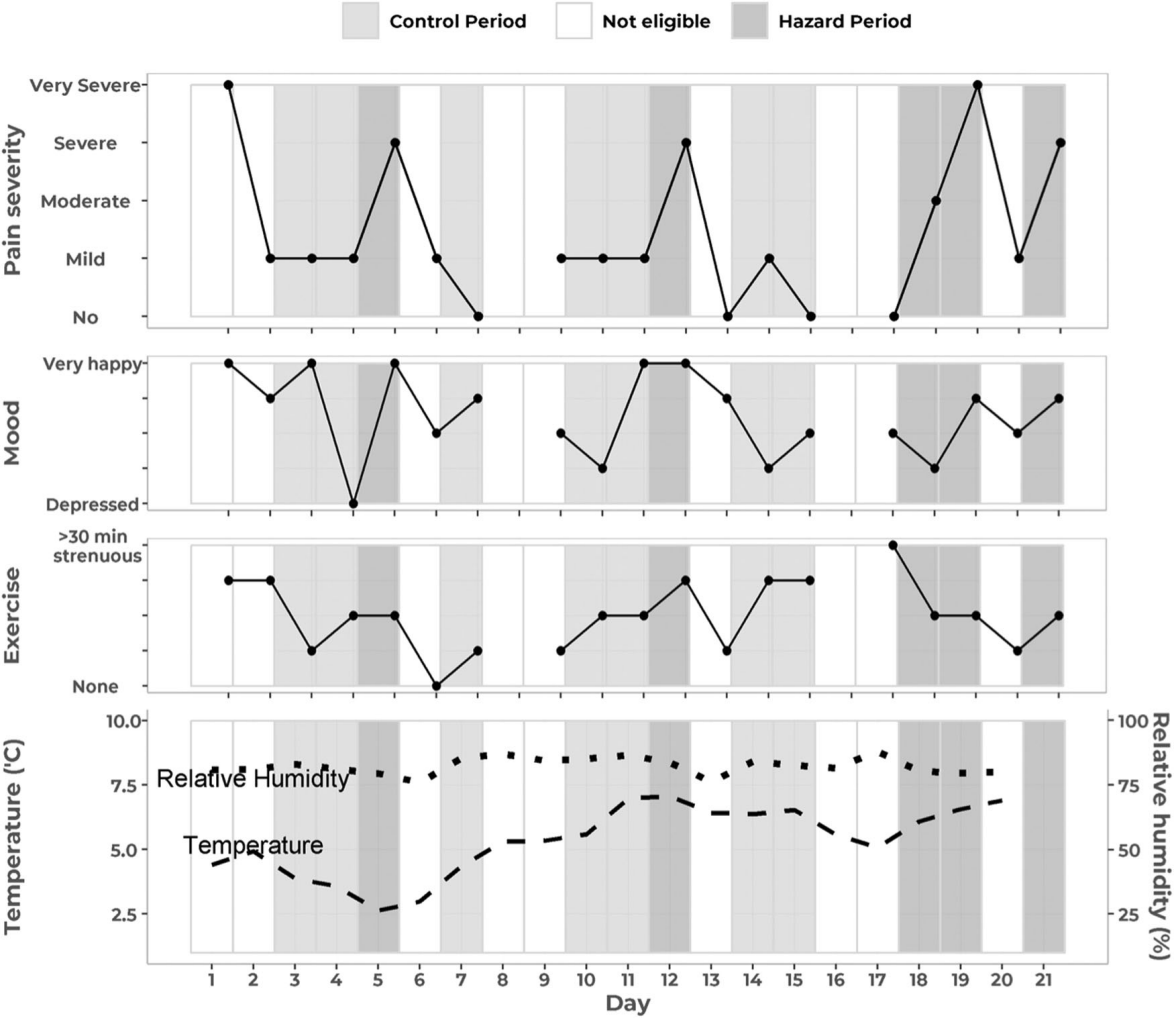
Example participant timeline of 21 days, showing participant-reported items (here, pain severity, mood, and exercise) and weather data (here, temperature and relative humidity). Pain events with their associated hazard periods (dark grey) occur when pain severity increases by two or more ordinal categories between consecutive days (e.g. from Day 4 to Day 5). Control periods (light gray) occur on days that were eligible to be a pain event, but where pain did not increase by two or more ordinal categories. Days where there was no recorded pain on the preceding day, or where the preceding day’s pain was severe or very severe (and could thus not increase by two or more categories), were not eligible to be pain-event days or control days. The case-crossover analysis compared the weather on pain-event days to weather on control days within a risk set of a calendar month

The study app was downloaded by 13,207 users over the 12-month recruitment period (Figs 1 and 2a) with recruitment from all 124 UK postcode areas. A total of 10,584 participants had complete baseline information and at least one pain entry, with 6850 (65%) participants remaining in the study beyond their first week and 4692 (44%) beyond their first month (Fig. 2b). Further description of engagement clusters is provided in Supplementary Table 2 and Supplementary Figs 1–3. A total of 2658 participants had at least one hazard period matched to a control period in the same month (Fig. 3) and were included in the final analysis. There were 9695 hazard periods included in the analysis for the final 2658 participants, matched to 81,727 control periods in 6431 participant-months. A total of 1235 participants contributed one month, and the remaining 1423 participants contributed 2–15 months.

**Fig. 4:**
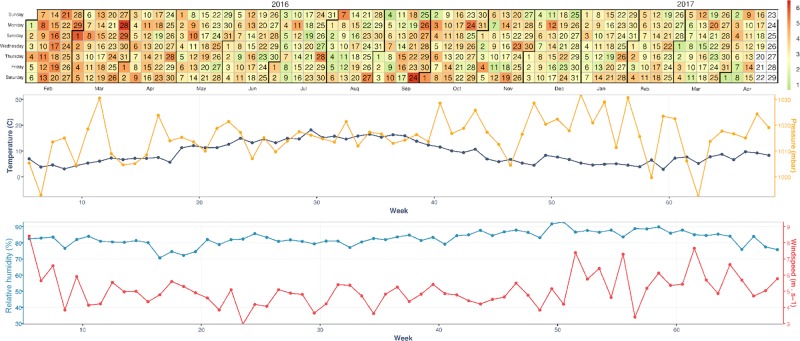
The proportion of eligible active participants reporting a pain event during the study period, aligned with average UK weather data from February 2016 to April 2017. Heat map colors indicate the percentage of participants reporting a pain event on that day, ranging from 1–6% participants. The denominator per day is the number of participants who reported their pain on the day of interest and the prior day, irrespective of the level of pain on the prior day and thus their eligibility for a pain event

The final cohort was active for a median of 165 days (interquartile range, IQR 84–245) with symptoms submitted on an average of 73% of all days. Cohort members were predominantly female (83%), had a mean age of 51 years (standard deviation 12.6), and had a range of different pain conditions, predominantly arthritis (Supplementary Table 1). The median number of weather stations associated with each participant during the course of their active data-collection period was 9 (IQR 4–14) with a maximum of 82 stations, indicating how mobile participants were during the course of the study and the importance of accounting for the weather at different locations over the course of the study. As an illustration of the structure of the data, the proportion of participants reporting a pain event was plotted as a heat map per calendar day for the study period (Fig. 4), aligned with the average United Kingdom weather data for the same time period. On any given day during the study, about 1–6% of participants had a pain event. At the start of the study, most participants believed in an association between weather and their pain (median score 8 out of 10, IQR 6–9). The demographics, health conditions and baseline beliefs of the 2658 participants included in the analysis were representative of the 10,584 participants who downloaded the app and provided baseline information (Supplementary Table 2).

### Weather and pain

The multivariable case-crossover analysis including the four state weather variables demonstrated that an increase in relative humidity was associated with a higher odds of a pain event with an OR of 1.139 (95% confidence interval 1.099–1.181) per 10 percentage point increase, as was an increase in wind speed with an OR of 1.02 (1.005–1.035) per 1 m s^−1^ increase (Table 1). The odds of a pain event was lower with an increase in atmospheric pressure with an OR of 0.962 (0.937–0.987) per 10-mbar increase. Temperature did not have a significant association with pain (OR 0.996 (0.985–1.007) per 1 °C increase). The odds of a pain event was 12% higher per one standard deviation increase in relative humidity (9 percentage points) (OR 1.119 (1.084–1.154), compared to 4% lower for pressure (OR 0.958 (0.930–0.989) and 4% higher for wind speed (OR 1.041 (1.010–1.073) (11 mbar and 2 m s^−1^, respectively). Of the four weather variables, relative humidity had the strongest association with pain, and temperature the least, evidenced by the estimated relative importance of the variables and their standardized odds ratios (Table 1, Supplementary Table 4). Similar effect sizes were seen when each variable was examined in univariable analyses. Precipitation was not associated with an increased odds of a pain event (OR 0.996 (0.989–1.003) per 1 mm daily rainfall amount) (Supplementary Table 5). Exploratory analyses considered time spent outside by including an interaction term with temperature, relative humidity, and wind speed. Time spent outside did not have a significant interaction with relative humidity or wind speed, nor did it lead to significant associations for temperature when conducting analyses stratified by time spent outside (Supplementary Table 3). It thus was not included in the final model.

**Table 1:** Association between weather and pain from the case-crossover analysis in 2658 participants

Variable	Univariable (single weather variable only) Odds ratio (95% CI)	Multivariable (all weather variables only) Odds ratio (95% CI)	Multivariable (weather plus activity and mood) Odds ratio (95% CI)
*Temperature*			
Per 1 °C	1.001 (0.991–1.012)	0.996 (0.985–1.007)	1.001 (0.989–1.013)
Per 1 s.d. (4.8 °C)	1.007 (0.956–1.060)	0.981 (0.929–1.035)	1.005 (0.949–1.064)
*Relative humidity*			
Per 10%	1.148 (1.108–1.189)	1.139 (1.099–1.181)	1.117 (1.075-1.16)
Per 1 s.d. (8.6%)	1.126 (1.092–1.61)	1.119 (1.084–1.154)	1.100 (1.064–1.136)
*Pressure*			
Per 10 mbar	0.936 (0.914–0.958)	0.962 (0.937–0.987)	0.966 (0.94-0.993)
Per 1 s.d. (11.1 mbar)	0.930 (0.905–0.955)	0.958 (0.930–0.986)	0.963 (0.934–0.992)
*Wind speed*			
Per 1 m s^–1^	1.023 (1.01–1.037)	1.02 (1.005–1.035)	1.011 (0.995–1.027)
Per 1 s.d. (2.1 m s^–1^)	1.048 (1.020–1.077)	1.041 (1.010–1.073)	1.022 (0.990–1.056)
*High activity*			0.939 (0.881–1.002)
*Low mood*			4.083 (3.824–4.360)

High activity—Top three categories: 30 min or more of light or strenuous activity per day, or less than 30 min of strenuous activity

Low mood—Bottom three categories: ‘depressed’, ‘feeling low’ or ‘not very happy’ *s.d*. standard deviation

Distribution of weather variables:

Temperature: range −4.9 to 25.9 °C, s.d. 4.8 °C

Relative humidity: range 43.8–100%, s.d. 8.6%

Pressure: range 966–1044.8 mbar, s.d. 11.1 mbar

Wind speed: range 0–21.5 m s^−1^, s.d. 2.1 m s^−1^

The model was then expanded to include mood and physical activity on the day of interest, included as binary variables (Table 1), resulting in a modest reduction in the point estimates for all weather variables. Mood was strongly and independently associated with pain events (OR 4.083 (3.824–4.360) for low mood versus good mood), whereas there was no significant association with physical activity (OR 0.939 (0.881–1.002) for high versus low activity).

**Fig. 5:**
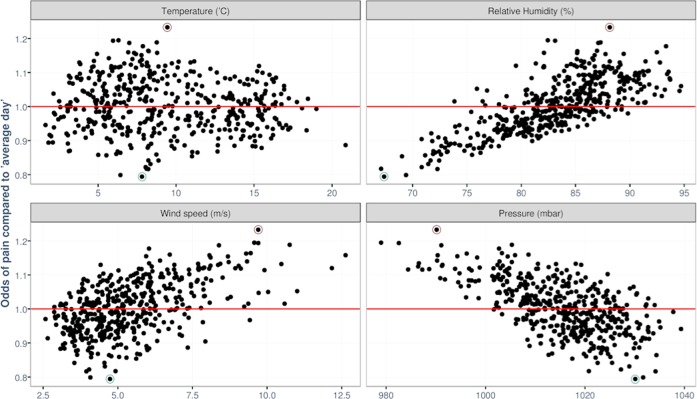
Estimated odds of a painful day for all weather days experienced during the 15 months. Estimated odds of a painful day are plotted as the odds ratio for each day compared to the average weather day in this period (temperature = 9.3 °C, relative humidity = 83%, wind speed = 4 m s^–1^ and pressure = 1013 mbar). Estimated odds are calculated from the output of the multivariable regression analysis. The day associated with the highest estimated odds of a pain event had a temperature of 9 °C, relative humidity 88%, wind speed 9.5 m s^–1^ and pressure 988 mbar. The day associated with the lowest estimated odds of a pain event was when the temperature was 7 °C, relative humidity was 67%, wind speed 4.5 m s^−1^ and pressure 1030 mbar

This multivariable regression model output represents the effect of one weather variable while all else remains constant. In reality, a single weather variable rarely changes in isolation while others remain unchanged. To illustrate the composite effect of the weather variables on the odds of reporting pain, an OR was calculated for each day using the coefficients of our multivariable model and daily UK mean weather values. Figure 5 demonstrates there is significant variability in the odds of a pain event for any given value of each weather variable. For example, at a temperature of 8 °C, the odds of a pain event varied from around 0.8–1.2, depending on the other state variables in the weather that day.

Other factors such as day of the week (Supplementary Table 6), lagged weather values (Supplementary Table 7) and changes in weather variables from the previous day were tested. Mondays, Thursdays, and Saturdays (ORs 1.14, 1.14, and 1.29, respectively) had higher odds of pain compared to Sundays, but adjusting for the day of the week did not alter the effect of the four main weather variables. Except for relative humidity (1-day lag and 2-day lag), no significant associations were observed between lagged weather variables and pain events. Including change in weather from yesterday showed a minor effect of changing relative humidity (OR 1.005 (1.001–1.009) per 10 percentage point increase), whereas the effects of today’s relative humidity and pressure remained unchanged (Supplementary Table 8). Stratification by disease led to a loss of statistical power and largely inconclusive results, although relative humidity appeared to have a stronger association with pain in patients with osteoarthritis (Supplementary Table 9, Supplementary Fig. 4). Stratification by the number of pain sites also showed no meaningful difference (Supplementary Table 10). After stratifying by participants’ prior beliefs about their weather–pain relationship, relative humidity remained associated with pain in all participants although the association with pressure was only seen in those with a strong prior belief (Supplementary Table 11).

## Discussion

This study has demonstrated that higher relative humidity and wind speed, and lower atmospheric pressure, were associated with increased pain severity in people with long-term pain conditions. The most significant contribution was from relative humidity. The effect of weather on pain was not fully explained by its day-to-day effect on mood or physical activity. The overall effect sizes, while statistically significant, were modest. For example, the ‘worst’ combination of weather variables would increase the odds of a pain event by just over 20% compared to an average day. Nonetheless, such an increased risk may be meaningful to people living with chronic pain.

In addition to investigating the weather–pain relationship, we successfully conducted a national smartphone study that delivered on the promise of how consumer technology can support health research.^12,15^ This study recruited over 10,000 participants throughout the United Kingdom, sustained daily self-reported data over many months,^13^ and showcased the value of passively collected GPS data. Prior large smartphone studies have retained only around one in ten participants for seven days or less.^16,17^ In contrast, our study retained 65% of participants for the first seven days, and 44% for the first month, with over 2600 participants contributing to the analysis having provided data for many months of the study.^13,14^ An important success factor was strong public involvement in early setup and piloting, as well as participants’ interest in weather as a possible pain trigger.^14^ The study design has resolved problems of prior weather–pain studies such as small populations,^5,7^ short follow-up,^3,8^ surrogate pain outcomes,^11^ the absence of possible causal pathway variables such as mood, and assumptions about where participants were located and thus the weather to which they were exposed.^18,19^ Overcoming these obstacles produced a large dataset that allowed us to tease out subtle relationships between weather and pain.

There are potential limitations to this study. First, the reduction in participant numbers from over 10,000 with baseline data to the final 2658 participants with at least one within-month risk set raises questions about generalisability. Importantly, the characteristics of those included in the analysis were similar to the initial 10,000 participants, other than being slightly older (mean age 51 versus 48 years old). In a prior analysis, we showed that Cloudy participants were largely representative of a population reporting chronic-pain symptoms,^13^ although proportionally fewer participants at both extremes of age were recruited. However, we would not expect middle-aged recruits to differ in their relationship between weather and pain from older or younger participants, and thus such selection factors would not invalidate our results. Second, the study was advertised to participants with a clear research question. It is possible that only people with a strong belief in a weather–pain relationship participated, generating an unrepresentative sample. However, the percentage of participants who believed in the weather–pain relationship was similar to prior studies,^20^ and we did not see selective attrition of people who reported no weather–pain beliefs.^13^ The within-person design would, regardless, mean that participants who drop out early would not introduce bias from time-invariant characteristics. Third, the lack of blinding raises possible information bias where observed weather could influence participants’ symptom reporting. Our baseline questionnaire demonstrated that rain and cold weather were the most common pre-existing beliefs. If a reporting bias were to exist, we would expect higher pain to be reported at times of colder weather. Our findings—including the absence of an association with either temperature or rainfall—cannot be explained by such a reporting bias. Fourth, pain reporting is subjective, meaning one participant’s “moderate” might equate to someone else’s “severe”. The within-person case-crossover analysis meant we compared moments when an individual’s score increased by a meaningful amount to a control period for that same person. Fifth, we chose to model the weather using daily averages. It is possible that other findings may be hidden if the association between weather and pain was with other metrics of weather, such as the daily maximum, minimum, or range, or even if the changes in weather on hourly time scales affect participants’ pain. Sixth, the findings from this United Kingdom study cannot necessarily be extrapolated to different climates where the weather is different. Seventh, our population-wide analysis assumed that all participants have the same weather–pain relationship. Different diseases may have different sensitivities to pain and, even within disease, participants may be affected differently. Our decision to use the whole chronic-pain population in our primary analysis means the overall associations with weather variables may be combinations of strong, weak and absent causal effects, thereby underestimating the most important associations. Notable differences were not seen after stratification by pain condition, although the power to detect any differences was reduced because of smaller sample sizes. Lastly, the inclusion of repeated events per person required us to consider within-subject dependence which, if not accounted for, would lead to bias.^21^ Our outcome was based on changes in pain (a two or more category increase), which meant events rarely occurred on consecutive days, thereby ensuring a time gap between recurrent events and the avoidance of bias.

Understanding the relationship between weather and pain is important for several reasons. First, this study validates the perception of those who believe that their pain is associated with the weather. Second, given we can forecast the weather days in advance, understanding how weather relates to pain would allow pain forecasts. Patients could then plan activities and take greater control of their lives. Finally, understanding the relationship between weather and pain might also allow better understanding of the mechanisms for pain and thus allow the development of new and more effective interventions for those who suffer with pain.

In summary, our large national smartphone study has successfully supported the collection of daily symptoms and high-quality weather data, allowing examination of the relationship between weather and pain. The analysis has demonstrated significant relationships between relative humidity, pressure, wind speed and pain, with correlations remaining even when accounting for mood and physical activity.

## Methods

### Patient involvement

Patient involvement has been important throughout the study, from inception to interpretation of the results. Co-author C.G. is a patient partner and co-applicant, while a patient and public involvement group of seven additional members has supported the study, meeting eight times in total. During the feasibility study,^14^ patients positively influenced the wording and display of questions within the app. C.G. and other members of the Patient and Public Involvement Group were involved in media broadcasts at study launch and subsequent public engagement activities, explaining why the research question was important to them and relevant to patients with long-term pain conditions.^22^ They have supported the interpretation of findings and the development of dissemination plans for the results, ensuring the results reach study participants, patient organizations and the general public.

### Recruitment

We recruited participants through local and national media (television, radio, and press) and social media from 20 January 2016 to 20 January 2017. To participate in the study, participants needed to (i) be living with long-term (>3 months) pain conditions, (ii) be aged 17 years or older, (iii) be living in the United Kingdom, and (iv) own an Android or Apple iOS smartphone. Interested participants were directed to the study website (www.cloudywithachanceofpain.com) where they could check their eligibility, learn about the study, and download the uMotif app (Fig. 1). After downloading the study app, participants completed an electronic consent form and a baseline questionnaire including demographic information (sex, year of birth, first half of postcode), anatomical site(s) of pain, underlying pain condition(s), baseline medication use, and beliefs about the extent to which weather influenced their pain on a scale of 0–10, including which weather condition(s) were thought to be most associated with pain. Participants were then invited to collect daily symptoms for six months, or longer if willing. Each day, the app alerted participants to complete ten items at 6:24 p.m. (Fig. 1). The ten items were pain severity, fatigue, morning stiffness, impact of pain, sleep quality, time spent outside, waking up feeling tired, physical activity, mood, and well-being. Each data item had five possible labeled ordinal responses. For example, in response to the question “How severe was your pain today?”, possible responses were “no pain”, “mild pain”, “moderate pain”, “severe pain” or “very severe pain”. The data were analysed using a case-crossover design where, for each participant, exposure during days with a pain event (“hazard periods”) were compared to “control periods” without a pain event in the same month.^23^ Pain events were defined as a two-or-more category increase in pain from the preceding day, consistent with more stringent definitions of a clinically important difference^24^ (Fig. 3). Data collection ended on 20 April 2017.

### Cohort selection

Participants were included in the final cohort for analysis if they fulfilled the following criteria: (1) downloaded the app; (2) provided consent; (3) completed the baseline questionnaire; and (4) contributed at least one pain event and matched control period in the same month (see below). During exploratory analysis, it was apparent that people reported higher pain levels in the first ten days following recruitment (perhaps due to calibration or regression to the mean). Therefore, the first ten days were excluded from the formal analysis. However, even if the first ten days were included, they had a negligible effect on the results (Supplementary Table 12).

The total person-days in study was calculated for each participant as the number of days between their first and last day of entering pain data. The number of person-days on which a pain score was entered was summed per participant, presented as a proportion of the total person-days in study, and averaged across the population. The geographical distribution of recruitment was described as the number of UK postcode areas represented (out of a maximum of 124).^25^ The movement of participants during the study was described as the median number of weather stations associated with each participant during their data-collection period.

### Ethical approval

Ethical approval was obtained from the University of Manchester Research Ethics Committee (ref: ethics/15522) and from the NHS IRAS (ref: 23/NW/0716). Participants were required to provide electronic consent for study inclusion. Further details are available elsewhere.^13,14^

### Weather data

Weather data were obtained by linking hourly smartphone GPS data to the nearest of 154 possible United Kingdom Met Office weather stations. Where GPS data were missing, we used significant location imputation. (For details, see supplement). Local hourly weather data were obtained from the Integrated Surface Database (ISD) of NOAA (http://www.ncdc.noaa.gov/isd), which includes hourly observations from UK Met Office weather stations.

Given the latitude–longitude coordinates of a participant location, the haversine distance to every Met Office weather station was calculated. The nearest station to the given location was selected, conditional on the distance being less than 100 km and the station having four weather variables (temperature, pressure, wind speed, and dewpoint temperature) available at that time. If all stations with the required weather data exceeded the maximum distance (100 km), the location was left unlinked and the observation was excluded from the analysis.

The significant location imputation approach for handling missing hourly GPS data had three stages.^26^ First, the participant’s observed location data were ordered by the frequency that the locations were visited. Second, the locations were spatially clustered using Hartigan’s Leader Algorithm^27^ with a threshold of 0.5 km. Third, missing locations during weekdays were replaced by the centroid of the participant’s most visited cluster for weekdays and missing locations during weekends were replaced by centroid of the participant’s most visited cluster for weekends.

### Recruitment and retention

Recruitment and duration of follow-up were presented as a graph of cumulative recruitment and active participants, with participation ending at the last symptom entry. Retention in the study was also presented as a survival probability against time since recruitment, with participants censored when they were no longer eligible for follow-up. Eligible follow-up time ranged from 90 days (for those recruited on 20 January 2017) to 456 days (for those recruited on 20 January 2016). Engagement of participants was further described through clustering of engagement states, which has been described in detail elsewhere.^13^ Following recruitment, individuals were labeled as engaged if they reported any of the ten symptoms on a given day. A first-order hidden Markov model was used to estimate the levels of engagement of participants by assuming three latent engagement states: high, low, and disengaged. Clusters were defined according to different probabilities of transitioning between high engagement, low engagement and disengagement during the study. Retention of active participants was also presented stratified by engagement cluster, and in the subset of participants who contributed to the final analysis.

### Analysis method

Days without pain events were only control periods if they were eligible to have a two-or-more category increase (i.e. the preceding day’s pain was lower than “severe”), thus fulfilling the exchangeability assumption for the case-crossover study design.^28^ With this design, participants serve as their own control, eliminating confounding by time-invariant factors. Each month per participant with at least one hazard and one control period formed a risk set. Conditional logistic regression was used to estimate the odds ratio (OR) for a pain event for four state weather variables (temperature, relative humidity, pressure, and wind speed). The condition logistic regression model was implemented with the assumption that the possible recurrent events (hazard periods) within a person are independent conditional on the subject-specific variables and other observed time-varying covariates. Further, we make sure that there is no overlap between case and control periods. Our assumption is reasonable given the time gap between subsequent events.

Each weather variable was included in univariable models and then all four were included in a multivariable analysis. Each weather variable was represented as a daily average per participant for the hazard or control period, with results presented as an OR for a pain event in response to a one-unit increase for temperature and wind speed (°C and meter per second, respectively) or a ten-unit increase for relative humidity and pressure (percentage points and millibar, respectively). Standardized odds ratios of each weather variable were also calculated. The relative importance of the four state weather variables was estimated by summing the Akaike weights.^29^ In all models, the preceding day’s pain score was included as it influenced the likelihood of a pain event the following day. The model was expanded to include mood and physical activity on the day of interest, included as binary variables. Time spent outside was considered as a possible effect modifier by including an interaction term with temperature, relative humidity, and wind speed. A directed acyclic graph is included in the supplementary material (Supplementary Fig. 5).

Sensitivity analyses were conducted to examine the effect of precipitation, day of the week, possible lag between weather and pain, change in weather from the day before the hazard or control day, disease type, sites of pain (single versus multiple sites) and prior beliefs in the weather–pain relationship. Respecting patients’ perspectives, we decided our primary analysis would focus on the whole chronic-pain population and our analyses of disease-specific associations would be secondary. We also re-ran the analysis including the first 10 days.

### Daily pain-event estimates

Estimated odds ratio for a pain event per day compared to the average weather day were calculated using the following equation:

$${\mathrm{Odds}}\,{\mathrm{Ratio}} = {\mathrm{exp}},\begin{array}{*{20}{l}} {\left\{ {{\mathrm{\beta }}_{\mathrm{T}}\left( {{\mathrm{temperature}} - {\mathrm{\mu }}_{\mathrm{T}}} \right) + {\mathrm{\beta }}_{{\mathrm{RH}}}\left( {\frac{{{\mathrm{relative}}\,{\mathrm{humidity}} \, - \, {\mathrm{\mu }}_{{\mathrm{RH}}}}}{{10}}} \right)} \right.} \hfill \\ {\left. { \,+\, {\mathrm{\beta }}_{{\mathrm{wsp}}}\left( {{\mathrm{wind}}\,{\mathrm{speed}} - {\mathrm{\mu }}_{{\mathrm{wsp}}}} \right) + {\mathrm{\beta }}_{\mathrm{P}}\left( {\frac{{{\mathrm{pressure}} \, - \, {\mathrm{\mu }}_{\mathrm{p}}}}{{10}}} \right)} \right\}} \hfill \end{array}$$

where

β_T_ = coefficient for temperature from final model,

β_RH_ = coefficient for relative humidity,

β_wsp_ = coefficient for wind speed,

β_P_ = coefficient for pressure, and

*μ*_*T*_ = mean temperature,

*μ*_*RH*_ = mean relative humidity,

*μ*_*wsp*_ = mean wind speed, and

*μ*_*p*_ = mean pressure

of the daily UK means over the study period.

The predicted odds ratios of a pain event, relative to the average weather day, were plotted for all days within our study period for each of the four state weather variables.

Statistical analyses were performed using R 3.3.0.^30^

### Reporting summary

Further information on research design is available in the Nature Research Reporting Summary linked to this article.

## Supplementary information

Supplementary materials

Reporting summary

## Data Availability

The datasets generated and analysed during the current study are available from the corresponding author on reasonable request.

## Code availability

Data management and analyses were performed in R 3.3.0. Code may be available on reasonable request.
