# Supplementary material for: How the weather affects the pain of citizen scientists using a smartphone app
Source: NPJ Digit Med. 2019 Oct 24;2:105. doi: 10.1038/s41746-019-0180-3 (PMC6811599; doi:10.1038/s41746-019-0180-3)
Supplement: Supplementary file 1 — Supplementary materials [file 41746_2019_180_MOESM1_ESM.pdf]

Supplementary Material for

How the Weather Affects the Pain of Citizen Scientists Using a Smartphone App

William G. Dixon, Anna L. Beukenhorst, Belay Birlie Yimer, Louise Cook, Antonio Gasparrini, Tal El-Hay, Bruce Hellman, Ben James, Ana M. Vicedo-Cabrera, Malcolm Maclure, Ricardo Silva, John Ainsworth, Huai Leng Pisaniello, Thomas House, Mark Lunt, Carolyn Gamble, Caroline Sanders, David M. Schultz, Jamie C. Sergeant, John McBeth

Correspondence to: will.dixon@manchester.ac.uk

Additional Results and Sensitivity Studies

Participants were clustered into four engagement states, labeled as high, medium, low and very low engagers (*13*). The numbers in each state, for those with baseline questionnaire data and at least one pain score, and for those in the case-crossover analysis, are shown in figure S1 with retention by engagement cluster shown in figure S2. Baseline data stratified by engagement state is shown in table S2.

Supplementary Figure 1. Flow-chart of eligibility from app downloads to final cohort, including information on engagement states at each step.

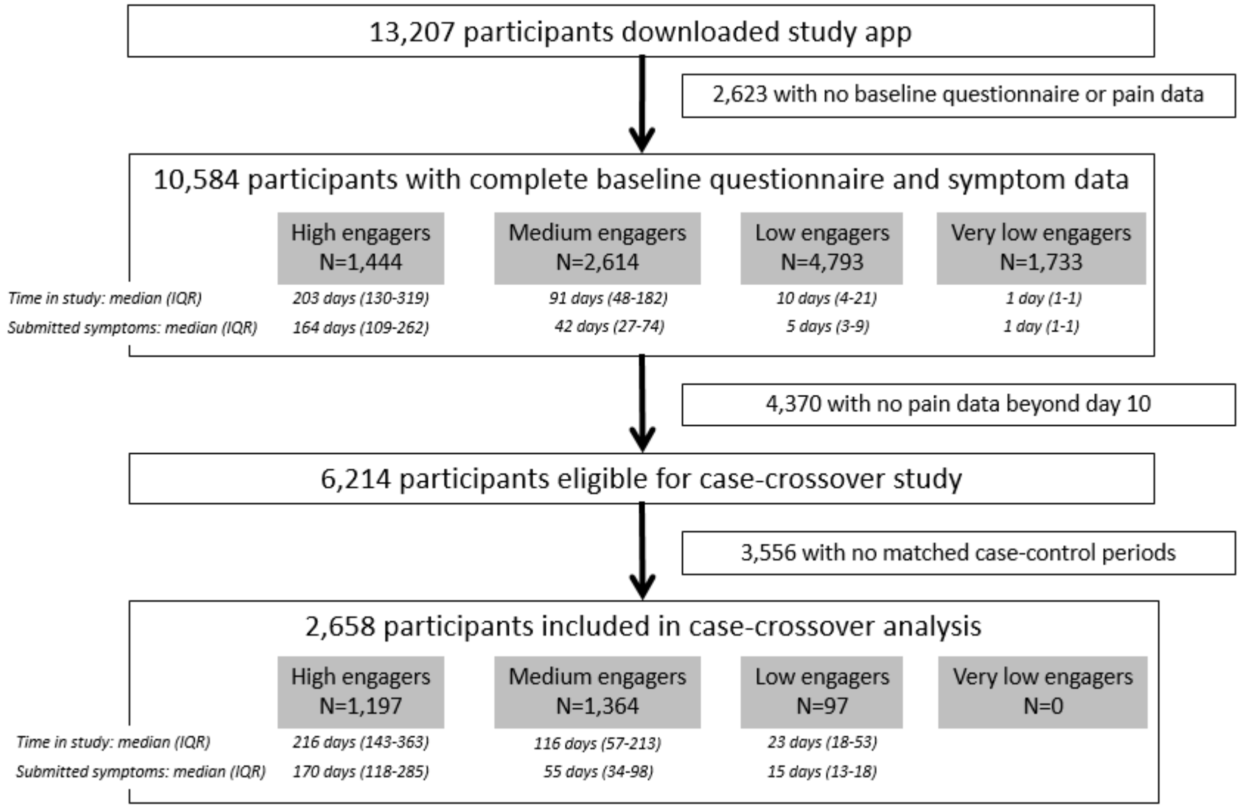

Supplementary Figure 2. Retention through time, stratified by engagement cluster. The graph represents the retention of active participants through time as a survival probability from the day of their recruitment, stratified by engagement cluster. Participants were censored when they were no longer eligible for follow-up. The shaded area represents the 95% confidence interval.

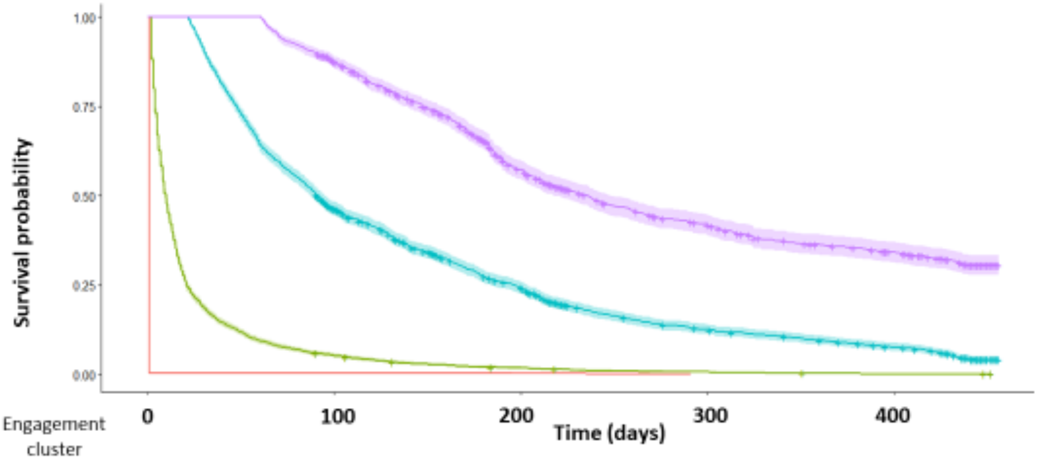

	Engagement cluster	0	100	200	300	400
Eligible for follow-up	High	1444	1389	1300	995	938
	Medium	2614	2511	2357	1559	1441
	Low	4793	4602	4358	3034	2842
	Very low	1733	1668	1593	1257	1193
Active Participants (% of eligible)	High	1444	1219 (88%)	741 (57%)	396 (40%)	299 (32%)
	Medium	2614	1174 (47%)	589 (12%)	240 (15%)	138 (10%)
	Low	4793	250 (5%)	82 (2%)	28 (1%)	5 (<1%)
	Very low	1733	6 (<1%)	4 (<1%)	0	0

Supplementary Figure 3. Retention through time for participants included in the final analysis cohort (n=2658). The graph represents the retention of active participants through time as a survival probability from the day of their recruitment, limited to the 2658 participants who were included in the case-crossover analysis. Participants were censored when they were no longer eligible for follow-up.

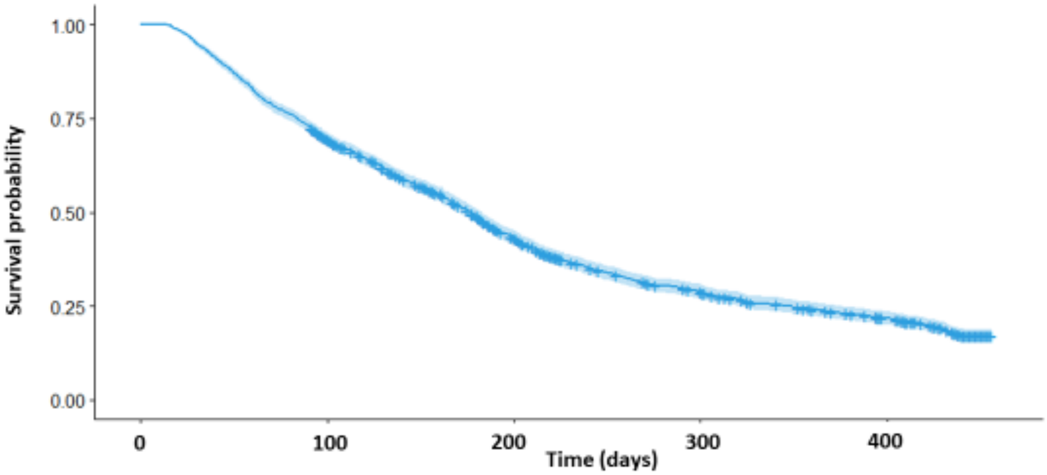

	0	100	200	300	400
**Eligible for follow-up**	2658	2573	2429	1802	1679
**Active Participants (% of eligible)**	2658	1793 (70%)	1059 (44%)	548 (30%)	384 (23%)

Supplementary Figure 4: Odds ratios for a pain event for each of the four state weather variable, stratified by disease (point estimates and 95% confidence intervals)

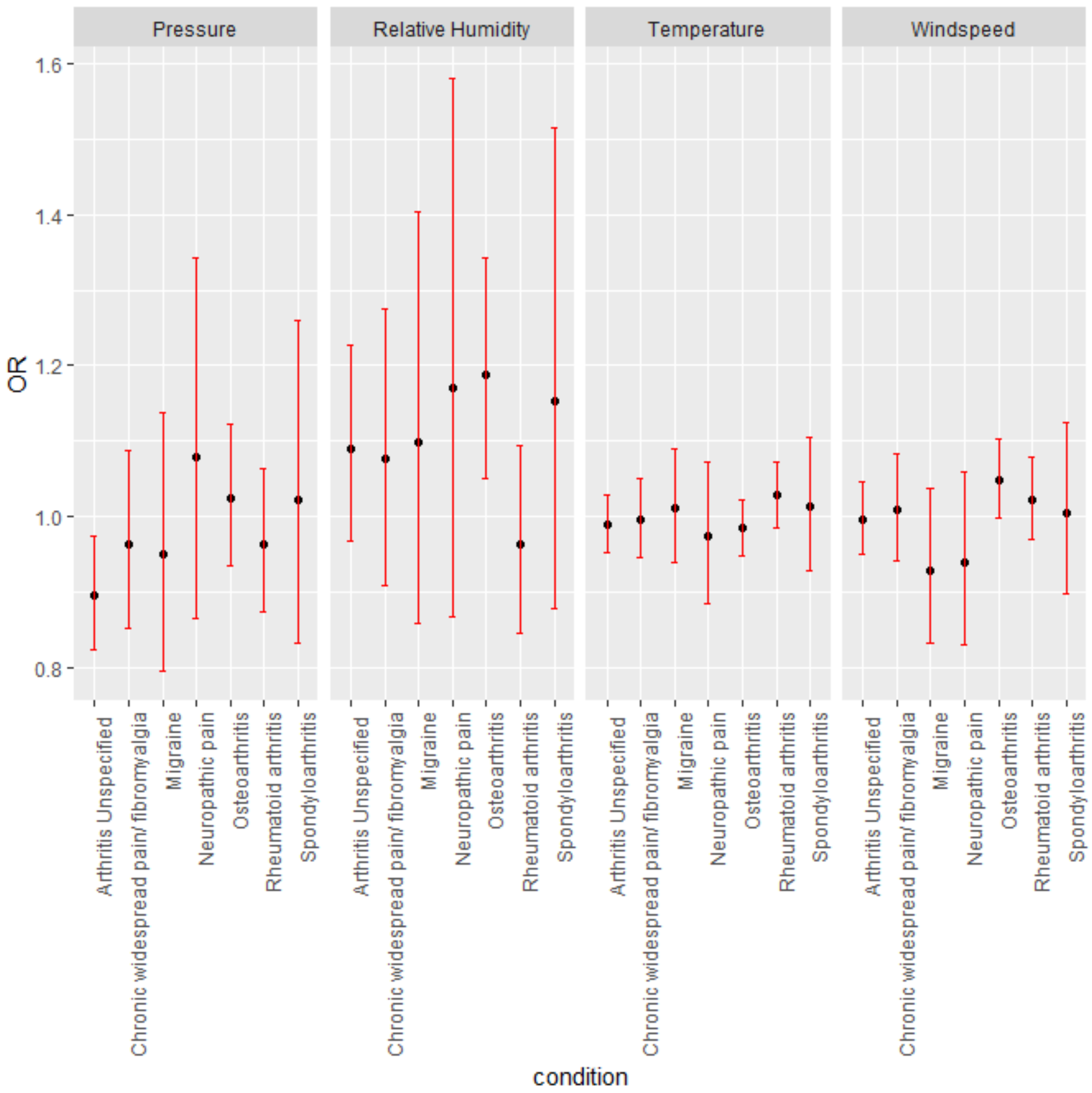

Supplementary Figure 5. Directed Acyclic Graph for the relationship between a pain event at day k (P), the four state weather variables (W), mood (M) and exercise (E).

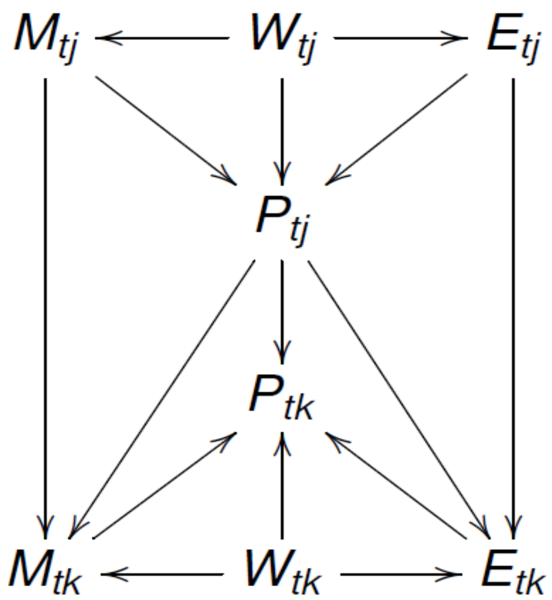

Figure S5 illustrates the relationship between a pain event at day k (P_{tk}), the four state weather variables (W), mood (M) and exercise (E). The likelihood of having a pain event today (P_{tk}) is influenced by today's weather (W_{tk}) directly as well as acting through mood (M_{tk}) and exercise (E_{tk}). Hence the path $W_{tk} \rightarrow P_{tk}$ is the direct effect of weather while $W_{tk} \rightarrow M_{tk} \rightarrow P_{tk}$ and $W_{tk} \rightarrow E_{tk} \rightarrow P_{tk}$ are the effect of weather mediated by mood and exercise, respectively. In order to estimate the direct effect we need to block the latter two. However, doing so opens path $W_{tk} \cdots > P_{tj}$ (pain event status in the previous day), since both variables are colliders. Hence, in the model used in this paper, we adjusted for P_{tj} which blocks this path in order to get the desired estimates. Adjusting for P_{tj} induces several paths between E_{tj} (exercise the previous day), M_{tj} (mood the previous day), and W_{tj} (weather the previous day), but all paths from these variables into P_{tk} will be blocked by P_{tj}. Time spent outside as a possible modifier is not included in the DAG

Supplementary Table 1. Baseline characteristics of study participants

	**Final case-crossover study cohort**	**Participants with complete baseline questionnaire and at least one pain recording**
Number of participants, *N*	2658	10584
**DEMOGRAPHICS**		
Female: *N* (%)	2210 (83.1)	8554 (80.8)
Age: mean (sd)	51.25 (12.6)	47.87 (13.2)
**DIAGNOSIS: *N* (%)**		
Rheumatoid arthritis	506 (19.0)	1954 (18.5)
Osteoarthritis	926 (34.8)	2552 (24.1)
Ankylosing spondylitis/ spondyloarthropathy	235 (8.8)	923 (8.7)
Gout	97 (3.6)	371 (3.5)
Arthritis (type not specified)	972 (36.6)	3662 (34.6)
Fibromyalgia/chronic widespread pain	665 (25.0)	2791 (26.4)
Chronic headache (including migraine)	271 (10.2)	1085 (10.3)
Neuropathic pain	371 (14.0)	1593 (15.1)
Other/no medical diagnosis	633 (23.8)	2758 (26.1)
**BELIEFS IN WEATHER-PAIN RELATIONSHIP: *N* (%)**		
Belief that weather influences pain on a scale of 0-10: median (IQR)	8 (6-9)	7 (6-9)
Weather conditions thought to be associated with pain		
Rain	1959 (73.7)	7705 (72.8)
Cold weather	1623 (61.1)	6941 (65.6)
Hot weather	458 (17.2)	1938 (18.3)
Changes in barometric pressure	1077 (40.5)	3687 (34.8)
Changes in temperature	825 (31.0)	3592 (33.9)
Other belief	125 (4.7)	351 (3.3)

Supplementary Table 2. Patterns of engagement for the 10,584 participants with completed baseline questionnaires and at least one pain recording.

	**Clusters of engagement (N = 10,584)**			
	**High**	**Medium**	**Low**	**Very Low**
Number of participants, *N*	1444	2614	4793	1733
Number of participants per engagement cluster included in the final case-crossover analysis	1197	1364	97	0
**ENGAGEMENT**				
Days in study<br>• Range<br>Median (IQR)	61-456<br>203<br>(130-319)	22-456<br>91<br>(48-182)	2-452<br>10<br>(4-21)	1-291<br>1<br>(1-1)
Number of days of pain data entry per individual<br>• Range<br>Median (IQR)	4-449<br>160<br>(104-254)	2-311<br>40<br>(26-72)	1-64<br>5<br>(2-9)	1-2<br>1<br>(1-1)
Proportion of total days on which pain data entered per individual:<br>median (IQR)	82<br>(73-89)	52<br>(38-67)	56<br>(32-81)	100<br>(100-100)
**DEMOGRAPHICS**				
Female: *N* (%)	1173 (81.2)	2182 (83.5)	3867 (80.7)	1332 (76.9)
Age: mean (sd)	54.0 (12.3)	48.7 (12.4)	46.0 (13.1)	46.5 (13.8)
**DIAGNOSIS: *N* (%)**				
Rheumatoid arthritis	284 (19.7)	496 (19.0)	870 (18.2)	304 (17.5)
Osteoarthritis	587 (40.7)	805 (30.8)	918 (19.2)	242 (14.0)
Ankylosing spondylitis/ spondyloarthropathy	129 (8.9)	230 (8.8)	424 (8.8)	140 (8.1)
Gout	49 (3.4)	100 (3.8)	154 (3.2)	68 (3.9)
Arthritis (type not specified)	570 (39.5)	872 (33.4)	1611 (33.6)	609 (35.1)
Fibromyalgia/chronic widespread pain	343 (23.8)	699 (26.7)	1336 (27.9)	413 (23.8)
Chronic headache (including migraine)	125 (8.7)	254 (9.7)	538 (11.2)	168 (9.7)
Neuropathic pain	206 (14.3)	414 (15.8)	736 (15.4)	237 (13.7)
Other/no medical diagnosis	293 (20.3)	656 (25.1)	1305 (27.2)	504 (29.1)
**BELIEFS IN WEATHER–PAIN RELATIONSHIP: *N* (%)**				
Belief that weather influences pain on a scale of 0–10: median (IQR)	7 (6-9)	8 (6-9)	7 (6-9)	7 (5-8)
Weather conditions thought to be associated with pain				

Rain	1064 (73.7)	1928 (73.8)	3519 (73.4)	1194 (68.9)
Cold weather	890 (61.6)	1657 (63.4)	3242 (67.6)	1152 (66.5)
Hot weather	257 (17.8)	464 (17.8)	922 (19.2)	295 (17.0)
Changes in barometric pressure	531 (36.8)	1023 (39.1)	1608 (33.5)	525 (30.3)
Changes in temperature	432 (29.9)	874 (33.4)	1686 (35.2)	600 (34.6)
Other belief	69 (4.8)	110 (4.2)	136 (2.8)	36 (2.1)

Supplementary Table 3: Case crossover analysis including an interaction term between time spent outside and temperature, relative humidity, and wind speed.

Model	Variable	OR (95% CI)
With temperature	Temperature	0.990 (0.977–1.004)
	Little time spent outside	1.035 (0.911–1.175)
	Interaction term	1.017 (1.005–1.030)
	Temperature, when little time spent outside	1.003 (0.980–1.027)
With relative humidity	Relative humidity	1.179 (1.113–1.249)
	Little time spent outside	1.698 (0.980–2.943)
	Interaction term	0.957 (0.896–1.023)
With wind speed	Wind speed	1.032 (1.007–1.057)
	Little time spent outside	1.298 (1.140–1.478)
	Interaction term	0.983 (0.956–1.011)

Supplementary Table 4. Standardized odds ratios and relative importance of weather variables in the case crossover analysis

**Predictor**	**Standardized Odds Ratio (95% CI)**	**Relative Importance (Summed Akaike weight*)**
Temperature (per sd increase)	0.981 (0.929–1.035)	0.32
Relative humidity (per sd increase)	1.119 (1.084–1.154)	1
Pressure (per sd increase)	0.958 (0.930– 0.986)	0.96
Wind speed (per sd increase)	1.041 (1.010– 1.073)	0.89

Multivariable model including the four state weather variables
*A variable that shows up in models with large weights will receive a high summed Akaike weight and is considered important.

Supplementary Table 5: Addition of precipitation to the univariable and multivariable case-crossover analysis results.

Model	Variable	Odds ratio (95% CI)
Univariable	Precipitation (per 1mm change)	1.005 (0.999–1.011)
Multivariable (weather only)	Temperature	0.995 (0.984–1.006)
	Relative humidity (per 10 percentage point change)	1.145 (1.103–1.188)
	Pressure (per 10 mbar change)	0.958 (0.932–0.985)
	Wind speed	1.021 (1.005–1.036)
	Precipitation	0.996 (0.989–1.003)
Multivariable, including mood and physical activity	Temperature	1.000 (0.988–1.012)
	Relative humidity (per 10 percentage point change)	1.120 (1.077–1.165)
	Pressure (per 10 mbar change)	0.963 (0.935–0.992)
	Wind speed	1.012 (0.996–1.028)
	Precipitation	0.996 (0.989–1.003)
	Low mood	4.046 (3.786–4.325)
	High activity	0.951 (0.891–1.015)

Supplementary Table 6: Inclusion of day of the week into the multivariable analysis including mood and activity.

**Variable**	**OR (95% CI)**
Temperature	1.000 (0.989–1.013)
Relative humidity<br>(per 10 percentage point change)	1.116 (1.074–1.16)
Pressure<br>(per 10 mbar change)	0.97 (0.944–0.998)
Wind speed	1.012 (0.996–1.028)
Low mood	4.093 (3.833–4.371)
High activity	0.937 (0.878–0.999)
Monday	1.139 (1.035–1.253)
Tuesday	1.094 (0.992–1.205)
Wednesday	1.037 (0.940–1.145)
Thursday	1.142 (1.035–1.261)
Friday	1.060 (0.959–1.171)
Saturday	1.292 (1.172–1.423)
Sunday	Referent

Supplementary Table 7. Effect of lagged weather variables on pain in the case crossover analysis. Weather days prior to the hazard/control period are added sequentially in single day increments into the multivariable model

Variable	Same day	Up to -1 day	Up to -2 days	Up to -3 days	Up to -4 days	Up to -5 days
**Temperature (hazard/control day)**	1.001 (0.989-1.013)	1.004 (0.988-1.020)	1.003 (0.987-1.020)	1.002 (0.986-1.019)	1.001 (0.985-1.018)	1.000 (0.984-1.017)
**Relative humidity (hazard/control day)**	1.117 (1.075-1.160)	1.141 (1.094-1.189)	1.142 (1.095-1.191)	1.143 (1.096-1.192)	1.145 (1.098-1.195)	1.144 (1.096-1.193)
**Pressure (hazard/control day)**	0.966 (0.940-0.993)	0.967 (0.924-1.012)	0.960 (0.914-1.009)	0.963 (0.916-1.012)	0.962 (0.915-1.011)	0.961 (0.914-1.010)
**Wind speed (hazard/control day)**	1.011 (0.995-1.027)	1.015 (0.997-1.034)	1.014 (0.996-1.033)	1.015 (0.997-1.034)	1.016 (0.997-1.034)	1.015 (0.997-1.034)
**High activity (hazard/control day)**	0.939 (0.881-1.002)	0.936 (0.877-0.998)	0.935 (0.877-0.998)	0.935 (0.877-0.998)	0.938 (0.879-1.001)	0.938 (0.879-1.001)
**Low mood (hazard/control day)**	4.083 (3.824-4.360)	4.070 (3.810-4.346)	4.061 (3.802-4.337)	4.067 (3.808-4.345)	4.072 (3.812-4.350)	4.079 (3.818-4.358)
**Temperature (preceding day)**	NA	0.998 (0.983-1.013)	1.000 (0.980-1.020)	1.001 (0.981-1.022)	1.003 (0.983-1.024)	1.004 (0.983-1.024)
**Relative humidity (preceding day)**	NA	0.995 (0.991-0.999)	0.994 (0.989-0.998)	0.993 (0.989-0.998)	0.993 (0.989-0.998)	0.993 (0.989-0.998)
**Pressure (preceding day)**	NA	0.999 (0.995-1.004)	1.001 (0.994-1.008)	1.000 (0.993-1.008)	1.000 (0.993-1.008)	1.001 (0.993-1.008)
**Wind speed (preceding day)**	NA	0.991 (0.973-1.009)	0.990 (0.970-1.010)	0.989 (0.969-1.010)	0.988 (0.968-1.008)	0.987 (0.967-1.008)
**Temperature (2 days ago)**	NA	NA	0.997 (0.981-1.012)	0.991 (0.971-1.012)	0.990 (0.970-1.011)	0.990 (0.969-1.010)
**Relative humidity (2 days ago)**	NA	NA	1.004 (1.000-1.008)	1.006 (1.001-1.010)	1.006 (1.001-1.010)	1.006 (1.001-1.010)
**Pressure (2 days ago)**	NA	NA	0.999 (0.994-1.004)	1.000 (0.992-1.007)	1.000 (0.992-1.008)	0.999 (0.991-1.007)

**Wind speed (2 days ago)**	NA	NA	1.004 (0.986-1.022)	1.006 (0.986-1.027)	1.006 (0.986-1.027)	1.006 (0.986-1.027)
**Temperature (3 days ago)**	NA	NA	NA	1.008 (0.993-1.024)	1.011 (0.991-1.032)	1.011 (0.991-1.033)
**Relative humidity (3 days ago)**	NA	NA	NA	0.997 (0.993-1.001)	0.996 (0.991-1.000)	0.996 (0.991-1.001)
**Pressure (3 days ago)**	NA	NA	NA	0.999 (0.994-1.004)	1.000 (0.992-1.007)	1.001 (0.993-1.009)
**Wind speed (3 days ago)**	NA	NA	NA	0.999 (0.981-1.017)	1.002 (0.982-1.022)	1.002 (0.982-1.022)
**Temperature (4 days ago)**	NA	NA	NA	NA	0.995 (0.979-1.010)	0.999 (0.979-1.019)
**Relative humidity (4 days ago)**	NA	NA	NA	NA	1.002 (0.998-1.006)	1.002 (0.998-1.007)
**Pressure (4 days ago)**	NA	NA	NA	NA	1.000 (0.995-1.005)	0.997 (0.990-1.005)
**Wind speed (4 days ago)**	NA	NA	NA	NA	0.994 (0.976-1.012)	0.992 (0.972-1.012)
**Temperature (5 days ago)**	NA	NA	NA	NA	NA	0.994 (0.979-1.009)
**Relative humidity (5 days ago)**	NA	NA	NA	NA	NA	0.999 (0.994-1.003)
**Pressure (5 days ago)**	NA	NA	NA	NA	NA	1.002 (0.997-1.007)
**Wind speed (5 days ago)**	NA	NA	NA	NA	NA	1.003 (0.985-1.021)

Supplementary Table 8. Inclusion of change in weather variable from the preceding day into the multivariable analysis including mood and activity.

**Variable**	**Odds ratio (95% CI)**
Temperature (per 1 °C change)	1.002 (0.988–1.015)
Relative humidity (per 10 percentage point change)	1.084 (1.034–1.136))
Pressure (per 10 mbar change)	0.959 (0.931–0.988)
Wind speed (per 1 ms^{-1} change)	1.006 (0.987–1.025)
Change in temperature (per 1 °C increase from yesterday)	1.002 (0.987–1.018)
Change in relative humidity (per 10 percentage point increase from yesterday)	1.005 (1.001–1.009)
Change in pressure (per 10 mbar increase from yesterday)	1.001 (0.996–1.005)
Change in wind speed (per 1 ms^{-1} increase from yesterday)	1.009 (0.991–1.027)
Low mood on hazard/control day	4.70 (3.810–4.346)
High activity on hazard/control day	0.936 (0.877–0.998)

Supplementary Table 9. Effect of temperature, pressure, humidity and wind speed, stratified by disease.

**Disease**	**Arthritis (unspecified)**	**Osteo-arthritis**	**Rheumatoid arthritis**	**Chronic widespread pain/ fibromyalgia**	**Neuro-pathic pain**	**Mig-raine**	**Spondylo-arthritis**
Temperature	0.990 (0.953-1.029)	0.985 (0.948-1.023)	1.028 (0.985-1.073)	0.996 (0.945-1.05)	0.973 (0.884-1.072)	1.012 (0.939-1.09)	1.013 (0.929-1.105)
Relative humidity	1.090 (0.967-1.228)	1.188 (1.05-1.344)	0.962 (0.845-1.095)	1.077 (0.908-1.276)	1.171 (0.866-1.582)	1.098 (0.858-1.404)	1.154 (0.878-1.516)
Pressure	0.896 (0.824-0.975)	1.024 (0.934-1.123)	0.964 (0.874-1.063)	0.963 (0.852-1.088)	1.078 (0.865-1.343)	0.951 (0.795-1.138)	1.023 (0.831-1.26)
Wind speed	0.996 (0.949-1.045)	1.049 (0.999-1.102)	1.023 (0.969-1.079)	1.009 (0.941-1.083)	0.938 (0.829-1.06)	0.929 (0.833-1.037)	1.005 (0.898-1.125)
High activity	0.943 (0.771-1.153)	1.130 (0.922-1.385)	0.860 (0.683-1.084)	0.792 (0.59-1.063)	1.120 (0.643-1.951)	1.061 (0.683-1.649)	0.968 (0.613-1.531)
Low mood	4.028 (3.257-4.983)	3.902 (3.16-4.819)	4.702 (3.765-5.873)	3.582 (2.696-4.759)	7.956 (4.595-13.775)	2.752 (1.871-4.048)	5.145 (3.237-8.178)
N participants	305	231	236	122	44	44	53

Analysis restricted to 1035 participants that were eligible for the case-crossover analysis *and* reported only one of these diagnoses; the remaining 1623 participants reported 2 or more of these conditions.

Supplementary Table 10. Effect of temperature, relative humidity, pressure and wind speed, stratified by number of self-reported sites of pain (single versus multiple)

Number of pain sites	Single site of pain	Multiple sites of pain
N participants	234	2424
Temperature	1.029 (0.99-1.069)	0.998 (0.986-1.011)
Relative humidity (per 10 percentage point change)	1.151 (1.019-1.301)	1.113 (1.069-1.159)
Pressure (per 10 mbar change)	0.957 (0.875-1.047)	0.967 (0.94-0.996)
Wind speed	1.017 (0.966-1.069)	1.010 (0.994-1.027)
High activity	1.005 (0.817-1.237)	0.933 (0.872-0.999)
Low mood	3.071 (2.488-3.792)	4.206 (3.925-4.508)

Supplementary Table 11. Effect of temperature, relative humidity, pressure and wind speed, stratified by prior belief.

Prior belief	Weak prior belief (0–6)	Strong prior belief (7–10)
N participants	828	1830
Temperature	1.006 (0.985-1.028)	0.999 (0.985-1.013)
Relative humidity (per 10 percentage point change)	1.143 (1.066-1.227)	1.106 (1.057-1.158)
Pressure (per 10 mbar change)	0.992 (0.943-1.043)	0.955 (0.924-0.987)
Wind speed	0.997 (0.969-1.026)	1.017 (0.998-1.036)
High activity	1.006 (0.896-1.129)	0.912 (0.844-0.986)
Low mood	3.826 (3.399-4.305)	4.204 (3.885-4.549)

Supplementary Table 12. Sensitivity analysis including all participants without follow-up restriction, alongside the final analysis restricting to those with 10+ days of data entry.

**Variable**	**Multivariable odds ratio (95% CI)**	
	**No restrictions:<br>1+ days data entry**	**Restricted to<br>10+ days data entry**
N	3309	2658
Temperature<br>• Per 1°C	0.999 (0.985-1.013)	1.001 (0.989-1.013)
Relative humidity<br>• Per 10 percentage points	1.108 (1.06-1.158)	1.117 (1.075-1.16)
Pressure<br>• Per 10 mbar	0.973 (0.942-1.005)	0.966 (0.94-0.993)
Wind speed<br>• Per $1ms^{-1}$	1.003 (0.985-1.022)	1.011 (0.995-1.027)
High activity	0.945 (0.877-1.018)	0.939 (0.881-1.002)
Low mood	4.074 (3.775-4.397)	4.083 (3.824-4.36)
